# The LcKNAT1-LcEIL2/3 Regulatory Module Is Involved in Fruitlet Abscission in Litchi

**DOI:** 10.3389/fpls.2021.802016

**Published:** 2022-01-21

**Authors:** Xingshuai Ma, Peiyuan Ying, Zidi He, Hong Wu, Jianguo Li, Minglei Zhao

**Affiliations:** ^1^State Key Laboratory for Conservation and Utilization of Subtropical Agro-Bioresources, Guangdong Laboratory for Lingnan Modern Agriculture, Key Laboratory of Biology and Genetic Improvement of Horticultural Crops (South China), Ministry of Agriculture and Rural Affairs, Guangdong Litchi Engineering Research Center, College of Horticulture, South China Agricultural University, Guangzhou, China; ^2^Guangdong Provincial Key Laboratory of Postharvest Science of Fruits and Vegetables, College of Horticulture, South China Agricultural University, Guangzhou, China

**Keywords:** litchi (*Litchi chinensis* Sonn.), fruitlet abscission, LcKNAT1, LcEIL2/3, ethylene

## Abstract

Large and premature organ abscission may limit the industrial development of fruit crops by causing serious economic losses. It is well accepted that ethylene (ET) is a strong inducer of organ abscission in plants. However, the mechanisms underlying the control of organ abscission by ET are largely unknown. We previously revealed that LcKNAT1, a KNOTTED-LIKE FROM *ARABIDOPSIS* THALIANA1 (KNAT1)-like protein, acted as a negative regulator in control of fruitlet abscission through suppressing the expression of ET biosynthetic genes in litchi. In this study, we further reported that LcKNAT1 could also directly repress the transcription of LcEIL2 and LcEIL3, two ETHYLENE INSENSITIVE 3-like (EIL) homologs in litchi, which functioned as positive regulators in ET-activated fruitlet abscission by directly promoting the expression of genes responsible for ET biosynthesis and cell wall degradation. The expression level of *LcKNAT1* was downregulated, while *LcEIL2*/*3* was upregulated at the abscission zone (AZ) accompanying the fruitlet abscission in litchi. The results of electrophoretic mobility shift assays (EMSAs) and transient expression showed that LcKNAT1 could directly bind to the promoters of *LcEIL2* and *LcEIL3* and repress their expression. Furthermore, the genetic cross demonstrated that the β-glucuronidase (GUS) expression driven by the promoters of *LcEIL2* or *LcEIL3* at the floral AZ was obviously suppressed by LcKNAT1 under stable transformation in *Arabidopsis*. Taken together, our findings suggest that the LcKNAT1-LcEIL2/3 regulatory module is likely involved in the fruitlet abscission in litchi, and we propose that LcKNAT1 could suppress both ET biosynthesis and signaling to regulate litchi fruit abscission.

## Introduction

Organ abscission is a widespread phenomenon throughout the life cycle of plants that occurs not only under stressful environmental conditions (biotic and abiotic) but also as a result of its own developmental process (Sexton and Roberts, 1982; Patterson, 2001; Taylor and Whitelaw, 2001; Roberts et al., 2002; Estornell et al., 2013). A high incidence of organ abscission (leaves, flowers, or fruits) may limit yield in crop species and, therefore, make their cultivation economically not viable. Thus, understanding the molecular mechanism of abscission is of great significance for the development of the crop industry.

Many factors coordinate the abscission process, of which plant hormones play a critical role. It is now well accepted that ethylene (ET) acts as a strong inducer in organ abscission. ET is formed from methionine *via* S-adenosyl-L-methionine (AdoMet) and 1-aminocyclopropane-1-carboxylic acid (ACC). ACC synthase (ACS) and ACC oxidase (ACO) catalyze the conversion of AdoMet to ACC and of ACC to ET, respectively (Yang and Hoffman, 1984). The effect of ET on organ abscission is closely related to its biosynthesis and signal transduction pathway. On the one hand, the increase of ET production was found in organs prior to abscission (Meir et al., 2019). On the other hand, organ abscission was induced or obviously accelerated after the application of exogenous ET, such as fruit abscission in mango (Nunez-Elisea and Davenport, 1986), olive (Kitsaki et al., 1999), apple (Dal et al., 2005), grape berry (Uzquiza et al., 2014), and litchi (Li et al., 2015; Ma et al., 2021), leaf abscission in citrus (Gomez-Cadenas et al., 1996), and flower abscission in *Lupinus luteus* (Wilmowicz et al., 2016). As the core downstream transcription factor (TF) of ET signaling, ETHYLENE INSENSITIVE 3 (EIN3) and EIN3-LIKE (EIL) proteins are considered to play a crucial role in ET-induced organ abscission. For example, *OeEIL2* was upregulated under ET treatment and was identified to be associated with mature fruit abscission in olive (Parra-Lobato and Gomez-Jimenez, 2011). ET treatment accelerated the leaf abscission of Chinese cabbage, in which the expression of *BcEIL1/2/3* was significantly induced (Meng et al., 2019). After knocking down the expression of *LeEILs*, the flower abscission was obviously reduced in tomatoes (Tieman et al., 2001). Recently, we also found that LcEIL2/3 functioned as positive regulators of fruitlet abscission in litchi, probably by activating the expression of genes responsible for ET biosynthesis and cell wall degradation (Ma et al., 2020). Taken together, previous studies strongly demonstrate that organ abscission is closely associated with ET biosynthesis and signaling. However, how the *ACS/ACO* genes are regulated and the mechanism underlying EIN3/EILs in the regulation of abscission remains elusive.

Litchi (*Litchi chinensis* Sonn.) is an important tropical and subtropical economic fruit crop. It is widely cultivated in over 20 countries. However, there are three or four waves of physiological fruit abscission dependent on cultivars throughout fruit development, leading to heavy economic loss and extremely limiting the development of the litchi industry (Yuan and Huang, 1988; Mitra et al., 2003). In *Arabidopsis*, KNOTTED-LIKE FROM *ARABIDOPSIS* THALIANA1/BREVIPEDICELLUS (KNAT1/BP) has been proven to be a key downstream factor of the INFLORESCENCE DEFICIENT IN ABSCISSION-HAESA/HAESA-LIKE2 (IDA-HAE/HSL2) pathway that controls the floral organ abscission in an ET-independent mode (Butenko et al., 2003; Cho et al., 2008; Stenvik et al., 2008; Shi et al., 2011; Santiago et al., 2016). In a previous study, we identified a KNAT1 homolog in litchi, i.e., LcKNAT1; interestingly, LcKNAT1 could suppress the ET production to control the fruitlet abscission by directly binding to the promoter of ET biosynthesis genes (Zhao et al., 2020). In this study, we further reported that LcKNAT1 could also directly bind to the promoters of *LcEIL2*/*3* and suppress their expression. These results suggest that LcKNAT1 could regulate the fruitlet abscission by suppressing both ET biosynthesis and signaling in litchi.

## Materials and Methods

### Plant Materials and Treatments

Three 20-year-old “Feizixiao” litchi trees (*L. chinensis* Sonn.) were randomly selected in the orchard of the South China Agricultural University. Twenty fruit-bearing shoots about 5–8 mm in different directions were chosen. Treatment of fruitlet removal at 25 days after anthesis was applied in ten shoots, and the other ten were used as control. Among each treatment, three shoots were used to monitor the dynamics of peduncle abscission and the others were used for sampling. Fruitlet abscission zone (FAZ) samples were collected and stored at -80°C for future analyses. Each tree was treated as a biological replicate (Li et al., 2015).

### Generation of Transgenic Plants

The coding sequence of *LcKNAT1* was cloned and infused into vector pCAMBIA1302 driven by the CaMV 35S promoter. Then, the recombinant construct was transformed into *Arabidopsis* Col plants according to the floral dip method (Clough and Bent, 1998). Transgenic lines were selected by hygromycin resistance and analyzed by quantitative reverse transcription PCR (qRT-PCR). T3 homozygous transgenic *Arabidopsis* plants were used for further analysis.

### 2′,7′-Bis-(2-Carboxyethyl)-5-(and-6)-Carboxyfluorescein Fluorescence Analyses

The 2′,7′-bis-(2-carboxyethyl)-5-(and-6)-carboxyfluorescein (BCECF) fluorescence analysis was carried out to measure cytosolic pH. According to the previously described studies (Sundaresan et al., 2014; Ying et al., 2016), *Arabidopsis* flowers were removed from the plant body and soaked into 10 μM BCECF-AM (B1150, Thermo Scientific) solution for 20 min under darkness. Then, phosphate-buffered saline (PBS, pH 7.4) was used to remove the excess BCECF-AM. Images were taken using the confocal laser scanning microscope (LSM 7 DUO, ZEISS, Germany). BCECF fluorescence and chlorophyll autofluorescence were detected under 488 nm and 633 nm light, respectively.

### Histochemical β-Glucuronidase Assays and Genetic Cross Assays

Notably, 1,500 bp promoter sequence of *LcEIL2* or *LcEIL3* was cloned and infused into vector pCAMBIA1391 which contains the β-glucuronidase (GUS) reporter, respectively. The recombinant constructs were transformed into *Arabidopsis* Col plants to obtain T3 homozygous *Pro_*LcEIL*2_:GUS* or *Pro_*LcEIL*3_:GUS* plants. Then, T3 homozygous *Pro_*LcEIL*2_:GUS* plants or *Pro_*LcEIL*3_:GUS* plants were crossed with T3 homozygous *35S:LcKNAT1-2* plants, and F1 *35S:LcKNAT1-2* × *Pro_*LcEIL*2_:GUS* plants or *35S:LcKNAT1-2* × *Pro_*LcEIL*3_:GUS* plants were used for GUS staining. In brief, flowers/siliques of transgenic plants were incubated in GUS staining buffer for 6 h at 37°C and then decolorized using 70% ethanol. GUS signals were detected by a stereoscope (ZEISS, SV11).

### Real-Time Polymerase Chain Reaction Analysis

Total RNA was extracted using the Column Plant RNAout Kit (TIANDZ, Beijing). The TransScript One-Step gDNA Removal Kit and the cDNA Synthesis SuperMix Kit (TransGen, Beijing) were used to generate the first-strand cDNA. qRT-PCR was performed using SYBR Green Polymerase Chain Reaction (PCR) SuperMix (Bio-Rad) on an ABI7500 Real-Time PCR System (Applied Biosystems). The expression level was analyzed using elongation factor 1-alpha (*EF-1a*) and *Ubiquitin 10* (*UBQ*) as an internal reference for litchi and *Arabidopsis*, respectively (Zhong et al., 2011; Ying et al., 2016). Three biological replicates were carried out.

### Yeast One-Hybrid Assay

Yeast one-hybrid (Y1H) assay was performed by using the Matchmaker Gold Yeast One-Hybrid System (Clontech). The coding sequence of LcKNAT1 was inserted into the pGADT7 vector. *LcEIL2* or *LcEIL3* promoter was cloned into the pAbAi vector. The recombinant pAbAi constructs were linearized and then transformed into the Y1H Gold strain, respectively. Positive yeast cells were transformed with pGADT7-LcKNAT1. The DNA–protein interaction was assessed based on the growth ability of transformed yeast on SD/-Leu medium with aureobasidin A (AbA).

### Electrophoretic Mobility Shift Assay

The sequence containing the DNA-binding domain of LcKNAT1 was cloned into vector PGEX-4T-1, and then, the recombinant construct was transformed into *Escherichia coli* strain BM Rosetta (DE3). The GST-LcKNAT1 fusion protein was purified using GSTSep Glutathione Agarose Resin (YEASEN, Shanghai). The probes including KNOX binding sites were biotin-labeled at the 3’ end, deriving from *LcEIL2* and *LcEIL3* promoters. The cold probes, containing the same sequences without biotin-labeled, and the mutant probes were used as competitors. These probes were incubated with GST-LcKNAT1 fusion protein. The DNA-binding assays were detected using the LightShift Chemiluminescent EMSA Kit (Thermo Scientific, Illinois, United States). ChemiDoc MP Imaging System (Bio-Rad, Hercules, CA, United States) was used to take the binding photos.

### Dual-Luciferase Reporter Assay

To detect the effect of LcKNAT1 on the transcriptional ability of *LcEIL2* and *LcEIL3*, a dual-luciferase (LUC) transient expression system was performed. The coding sequence of LcKNAT1 infused into pGreenII 62-SK vector was used as the effector. The promoter sequence of *LcEIL2* or *LcEIL3* inserted into pGreenII 0800-LUC vector was used as the reporter. Each pair of effector and reporter was incubated and co-transformed into tobacco leaves as reported previously (Ma et al., 2019). Notably, 2 days later, the Dual-Luciferase Assay reagents (Promega) were used to detect the LUC and renilla (REN) LUC activity, and then, the ratio of LUC/REN was calculated. For each pair, at least six independent replicates were performed.

### Statistical Analysis

Data were reported as the means ± SE. Statistical significance was detected according to the one-way ANOVA using SPSS (version 21.0, Chicago, IL, United States).

## Results

### *LcKNAT1* Is Decreased but *LcEIL2*/*3* Are Increased at Abscission Zone Accompanying the Fruitlet Abscission Process in Litchi

**FIGURE 1:**
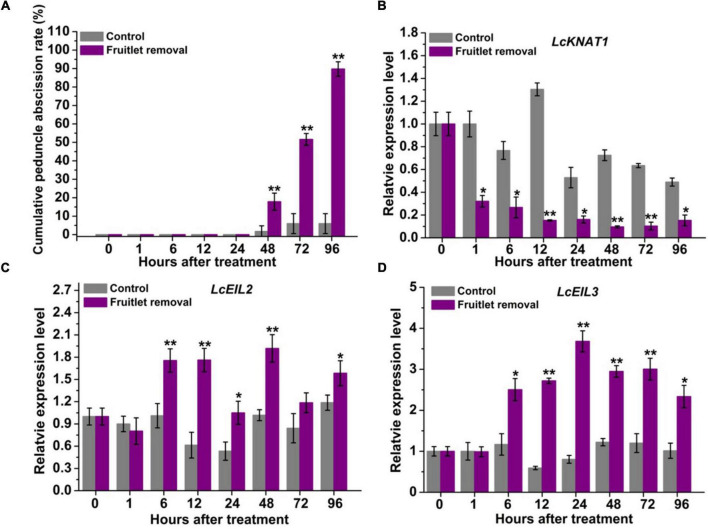
*LcKNAT1* is decreased, but *LcEIL2*/*3* expression is increased in the abscission zone (AZ) during the fruitlet removal-induced abscission process in litchi. **(A)** Fruitlet removal induced the peduncle abscission. The expression patterns of *LcKNAT1* **(B)**, *LcEIL2* **(C)**, and *LcEIL3* **(D)** after fruitlet removal. SE comes from three replicates. Asterisks indicate a significant difference (Student’s *t*-test: *P < 0.05, **P < 0.01).

Previously, *LcKNAT1* was found to be significantly downregulated, while *LcEIL2/3* was obviously upregulated after two fruitlet abscission-induced approaches, namely, girdling plus defoliation and ET treatments (Ma et al., 2020). To obtain more detailed information regarding their expression patterns, we performed fruitlet removal treatment in this study, and their expression was monitored starting from 1 h after treatment. As shown in Figure 1A, peduncle abscission was induced 48 h after treatment, possibly due to the continuous polar flow of auxin passing through the peduncle AZ was eliminated by the removal of the fruitlet (Meir et al., 2010; Kućko et al., 2020). At 96 h after fruitlet removal, more than 89% of peduncles were abscised. Consistent with our previous results, *LcKNAT1* was sharply reduced from 1 h after fruitlet removal and then maintained this low expression level throughout the abscission process (Figure 1B). In contrast, both *LcEIL2* and *LcEIL3* were significantly increased from 6 h after fruitlet removal (Figures 1C,D). Together, these results indicate that the expression pattern of *LcEIL2/3* is opposite to that of *LcKNAT1* during the fruitlet abscission process in litchi.

### LcKNAT1 Binds to the Promoters of *LcEIL2*/*3* and Represses Their Expression

**FIGURE 2:**
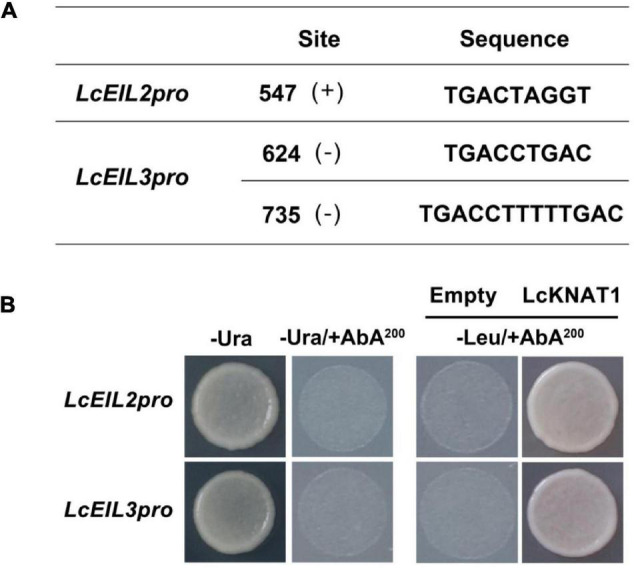
Binding of LcKNAT1 to the promoters of *LcEIL2* and *LcEIL3* by Yeast one-hybrid (Y1H) assay. **(A)** The core TGAC *cis*-elements were responsible for KNOX protein binding in the promoters of *LcEIL2* and *LcEIL3.* **(B)** The Y1H analysis of LcKNAT1 binding to *LcEIL2/3* promoters. Left: No basal activities of LcEIL2 or LcEIL3 promoter were detected in yeast grown on SD medium lacking Ura in the presence of aureobasidin A (AbA). Right: Yeast growth assay after the Y1H reporter strains were co-transformed with LcKNAT1 effector. The interaction was evaluated based on the growth conditions of transformed yeast on SD medium lacking Leu in the presence of AbA.

**FIGURE 3:**
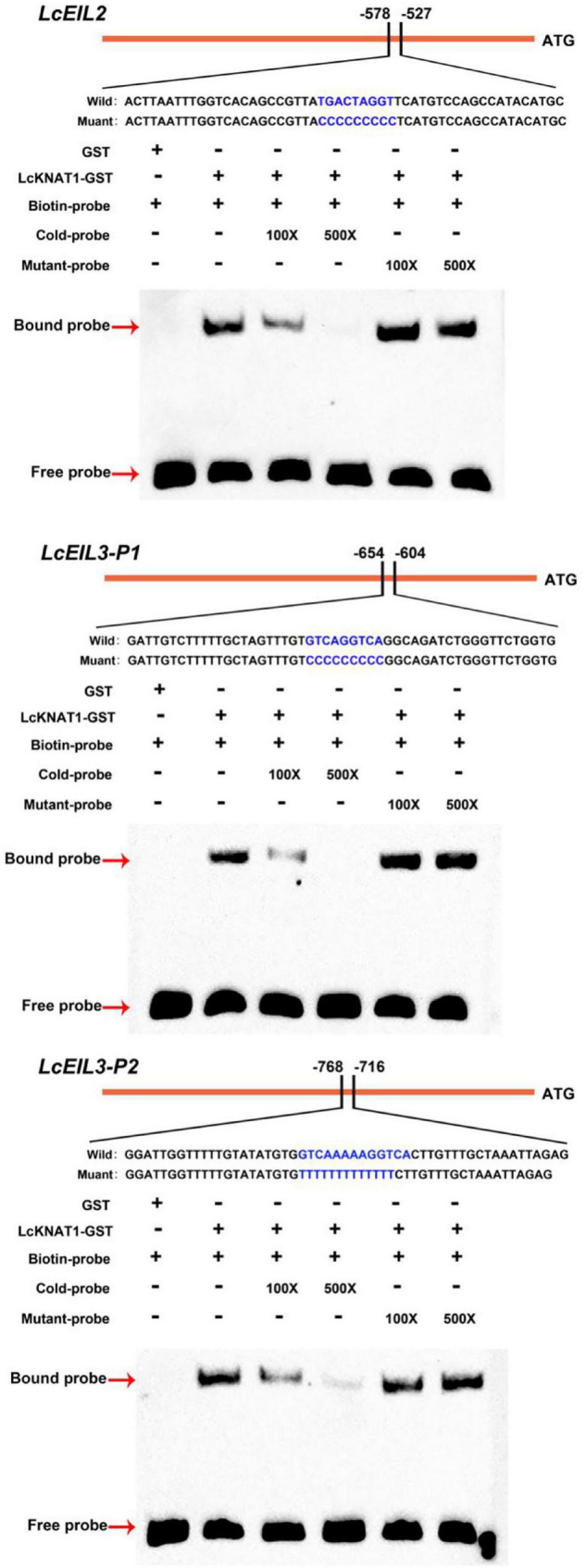
Binding of LcKNAT1 to *LcEIL2* and *LcEIL3* promoters *in vitro*. The electrophoretic mobility shift assay (EMSA) using purified LcKNAT1-GST fusion protein. The sequences of wild and mutant probes were shown at the top of the images. Shifted bands, suggesting the formation of DNA-protein complexes, are indicated by arrows. “ – ” represents absence, and “ + ” represents presence. 100 × and 500 × indicate the increasing concentrates of probes. Non-biotin-labeled probe with the same sequence was used as a cold competitor. Only the GST protein was served as a negative control.

Through screening the *cis*-elements in the promoter sequences of *LcEIL2*/*3*, we found that both *LcEIL2* and *LcEIL3* promoters contained the TGAC motifs, a KNOX TF-binding motif (Bolduc et al., 2012; Figure 2A). Thus, these findings prompted us to examine whether *LcEIL2*/*3* promoters could be recognized by LcKNAT1. To test it, we first performed a Y1H assay using the LcEIL2/3 promoters as baits and LcKNAT1 as prey. As shown in Figure 2B, the yeast cells containing the promoter of *LcEIL2* or *LcEIL3* failed to grow on SD/-Leu medium with AbA. In contrast, the yeast cells co-transformed with LcKNAT1, and the promoters of *LcEIL2* or *LcEIL3* could grow well on SD/-Leu medium with AbA, suggesting that LcKNAT1 can bind to the promoters of *LcEIL2* or *LcEIL3* and activate the reporter genes in yeast. We then carried out electrophoretic mobility shift assays (EMSAs) using GST-LcKNAT1 fusion protein. DNA fragments (∼50 bp) containing KNOX *cis*-element in the promoter regions of *LcEIL2*/*3* were synthesized and labeled with biotin at the 3′ end. As shown in Figure 3, when GST-LcKNAT1 fusion protein was incubated with labeled probes, the recombinant LcKNAT1 was able to strongly bind to the *LcEIL2* or *LcEIL3* promoter fragments, and this binding could be abolished by high concentrates of unlabeled competitors containing the same sequences in a dosage-dependent manner but not by competitors containing the mutant binding sites. These findings indicate that LcKNAT1 directly and specifically binds to the TGAC element within the promoter of *LcEIL2*/*3 in vitro*.

**FIGURE 4:**
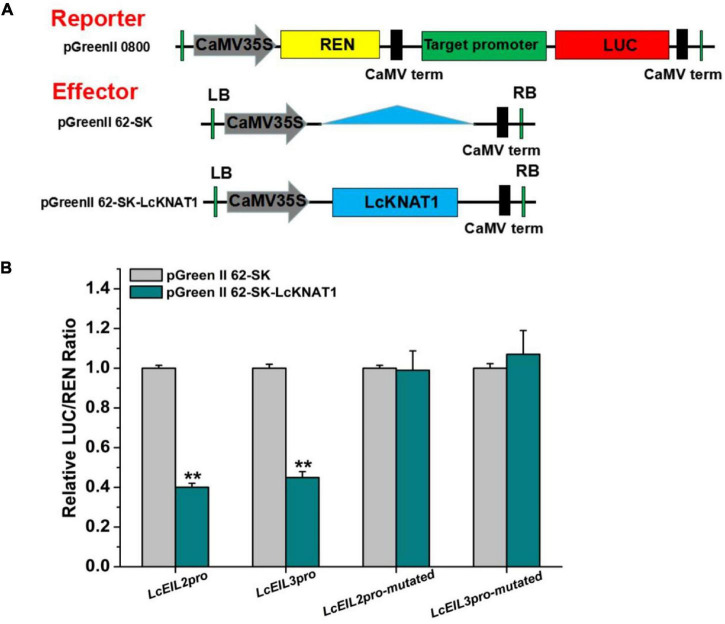
LcKNAT1 represses the transcriptional activity of *LcEIL2* and *LcEIL3*. **(A)** Schematic representation of the double reporter and effector plasmids used for LcKNAT1 transcriptional activity assay on target gene promoters. **(B)** Transient expression assays on LcKNAT1 transcriptional repression of *LcEIL2* and *LcEIL3*. The reporter and effector vectors were co-introduced into tobacco leaves. After 48-h incubation, the suppression of *LcEIL2/3* and mutated *LcEIL2/3* promoters by LcKNAT1 was shown by the ratio of LUC/REN. Mutated *LcEIL2/3* promoters were as shown in Figure 3. Each value represents the means of six biological replicates, and vertical bars represent ± SD. Asterisks indicate a significant difference (Student’s *t*-test: **P < 0.01).

Furthermore, we performed a dual-LUC assay to examine whether LcKNAT1 could repress the activity of *LcEIL2*/*3* promoters. As shown in Figure 4, compared with control, the LUC/REN ratio was dramatically reduced when either the *Pro_*LcEIL*2_*-LUC or the *Pro_*LcEIL*3_*-LUC reporter construct was co-transfected with the effector of pGreenII 62-SK-LcKNAT1, indicating that LcKNAT1 could repress the promoter activity of *LcEIL2*/*3*. Additionally, this repression was abolished when the core TGAC elements of *LcEIL2*/*3* promoters were mutated, indicating that LcKNAT1 can bind to the promoters of *LcEIL2*/*3 via* the core TGAC element and repress their activity.

### The Ectopic Expression of *LcKNAT1* in *Arabidopsis* Delays the Floral Organ Abscission and Suppresses the Promoter Activity of *LcEIL2*/*3*

**FIGURE 5:**
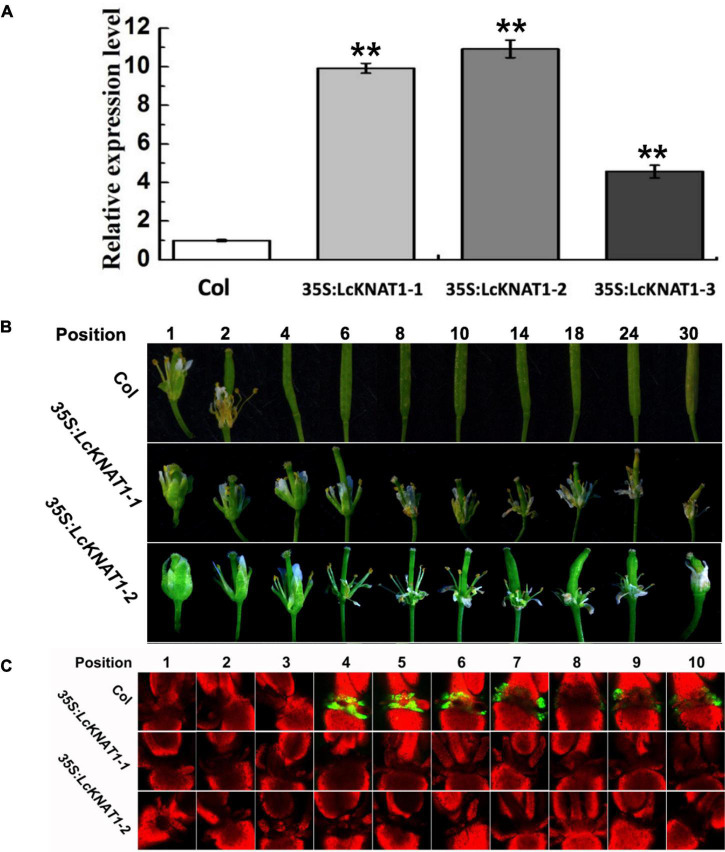
Ectopic expression of *LcKNAT1* in *Arabidopsis* suppresses floral organ abscission. **(A)** The expression levels of *LcKNAT1* in *Arabidopsis* transgenic lines. *LcKNAT1* driven by the CaMV 35S promoter was transformed into wild-type *Arabidopsis*. **(B)** Phenotypes of floral organ abscission process in Col and *LcKNAT1* transgenic lines. Floral organ position numbers were counted from the first flower with visible white petals on the top of the inflorescence. **(C)** BCECF fluorescence micrographs of floral organ AZ in Col and *LcKNAT1* transgenic lines. Inflorescence samples were separated from the plant body, incubated in BCECF-AM staining, and then examined using the confocal laser scanning microscope. Merged images of BCECF fluorescence with chlorophyll autofluorescence were shown, which are representative images out of 3–4 replicates. Asterisks indicate a significant difference (Student’s *t*-test: **P < 0.01).

To further confirm that *LcEIL2/3* is negatively regulated by LcKNAT1, we carried out a stable transformation assay in *Arabidopsis.* First, we generated three transgenic *Arabidopsis* lines expressing *LcKNAT1* (Figure 5A). *35S:LcKNAT1-1* and *35S:LcKNAT1-2* transgenic lines showed a higher expression level and were selected for further functional analysis. As shown in Figure 5B, for wild-type Col, when plants bore 30 siliques, there were only two siliques with floral organs attached. In contrast, *35S:LcKNAT1-1* and *35S:LcKNAT1-2* transgenic plants still retained the floral organs at position 30. The increase of cytosolic pH at AZ was reported to be closely associated with organ abscission in *Arabidopsis* (Sundaresan et al., 2014). We then used a pH-sensitive indicator, BCECF-AM, to detect the pH value in the cytosol of AZ tissues of transgenic plants. We found that BCECF signals were obviously appeared at position 4 in Col, while the BCECF signals could not be detected in both *35S:LcKNAT1-1* and *35S:LcKNAT1-2* transgenic lines, consistent with their floral organ abscission process (Figure 5C). Taken together, these findings suggest that LcKNAT1 functions as a strong negative regulator in control of the floral organ abscission in *Arabidopsis*.

**FIGURE 6:**
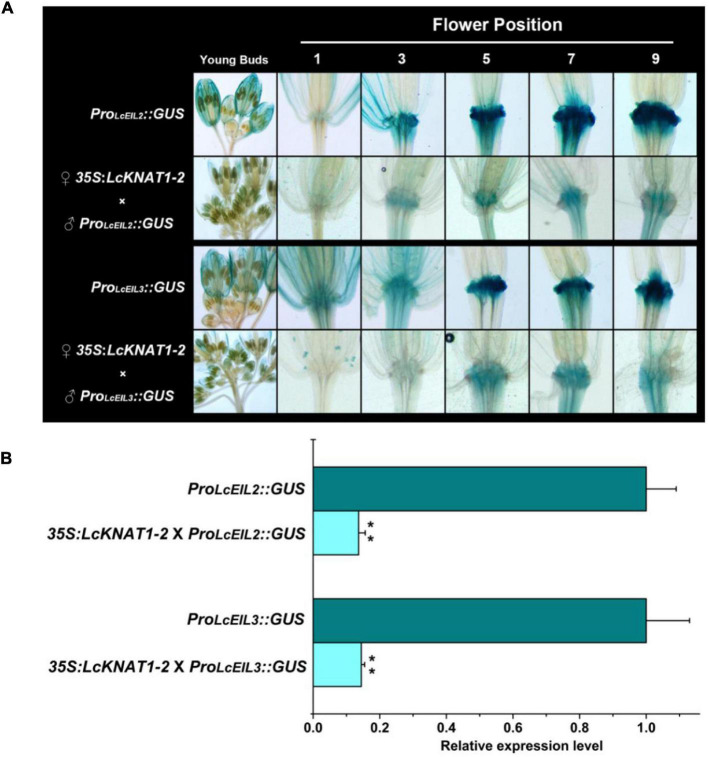
GUS expression driven by the promoter of *LcEIL2* or *LcEIL3* is repressed by LcKNAT1. **(A)** ♂*Pro_*LcEIL*2_:GUS* × ♀*35S:LcKNAT1-2* indicates that T3 homozygous *Pro_*LcEIL*2_:GUS* plant was crossed with T3 homozygous *35S:LcKNAT1-2* plant, and F1 *35S:LcKNAT1-2* × *Pro_*LcEIL*2_:GUS* or *35S:LcKNAT1-2* × *Pro_*LcEIL*3_:GUS* plants were used for GUS staining. **(B)** Quantitative assay of the GUS expression level in *Pro_*LcEIL*2_:GUS* plants, *35S:LcKNAT1-2* × *Pro_*LcEIL*2_:GUS* plants, *Pro_*LcEIL*3_:GUS* plants, and *35S:LcKNAT1-2* × *Pro_*LcEIL*3_:GUS* plants, respectively. Asterisks indicate a significant difference (Student’s *t*-test: **P < 0.01).

Then, we ectopically expressed the GUS reporter driven by the promoter of *LcEIL2* or *LcEIL3* in *Arabidopsis*. GUS staining revealed that both *LcEIL2* and *LcEIL3* had a dominant accumulation at the floral AZ. A stronger GUS signal in floral AZ could be detected from positions 3 to 9 in the *Pro_*LcEIL*2_:GUS* plant and positions 5 to 9 in *Pro_*LcEIL*3_:GUS* plant, respectively. Then, homozygous *Pro_*LcEIL*2_:GUS* plants or *Pro_*LcEIL*3_:GUS* plants were crossed with homozygous *35S:LcKNAT1-2* plants, and F1 *35S:LcKNAT1-2* × *Pro_*LcEIL*2_:GUS* plants or *35S:LcKNAT1-2* × *Pro_*LcEIL*3_:GUS* plants were used for GUS staining. As shown in Figure 6A, both the GUS signals driven by the promoter of *LcEIL2* or *LcEIL3* at the positions detected were significantly suppressed in the presence of *LcKNAT1*. Additionally, relative quantitative assays show that the expression level of the *GUS* gene driven by *LcEIL2* or *LcEIL3* promoter was obviously downregulated by *LcKNAT1*, with 7.3-fold and 6.9-fold reduction, respectively (Figure 6B), further supporting that LcKNAT1 could suppress the promoter activity of *LcEIL2*/*3 in planta*.

## Discussion

It is well known that ET is a strong inducer of organ abscission and has been widely reported in many fruit crops (Nunezelisea, 1986; Rasori et al., 2002; Dal et al., 2005; Gil-Amado and Gomez-Jimenez, 2012; Uzquiza et al., 2014; Li et al., 2015; Merelo et al., 2017). However, the mechanisms underlying ET in the regulation of abscission are largely unknown. In a previous study, we revealed that LcKNAT1 acts as a negative regulator of fruitlet abscission by repressing ET biosynthetic genes (Zhao et al., 2020). In this study, we further proved that the ET signaling components *LcEIL2/3* were also repressed directly by LcKNAT1 using both transient expression systems and stable transformation assays. We thus propose that a LcKNAT1-LcEIL2/3 regulatory module might be involved in the fruit abscission in litchi.

**FIGURE 7:**
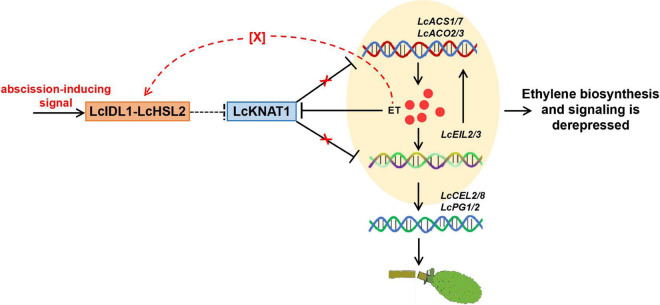
Proposed model for the role of LcKNAT1 in ethylene (ET)-regulated fruitlet abscission in litchi. LcKNAT1 can directly repress *LcACS1*/*7*, *LcACO2*/*3*, and *LcEIL2*/*3*, when LcKNAT1 is repressed by unknown abscission-inducing signals *LcACS1*/*7* and *LcEIL2*/*3* which are derepressed. Furthermore, LcEIL2/3 can also bind to *LcACS1*/*7* and *LcACO2*/*3* but promote their transcription, therefore forming a feedback loop to enhance the ET biosynthesis and signaling. Additionally, ET could also disable the repressor function of LcKNAT1 through the indirect activation of the LcIDL1-LcHSL2 module *via* an unknown factor, thereby enhancing the activation of the cell wall degradation genes *LcCEL2*/*8* and *LcPG1*/*2* to induce fruitlet abscission.

Results from the ectopic expression of *LcKNAT1* in tomatoes and *Arabidopsis* implied that LcKNAT1 likely played critical roles in the control of the fruitlet abscission in litchi. In combination with the findings from our previous and this study, how LcKNAT1 functions as a strong repressor during the fruitlet abscission in litchi could be partially explained as follows: (i) ET is the key inducer of abscission, and *LcACS1/7* and *LcACO2/3* involved in ET synthesis are induced at the fruitlet AZ; (ii) LcKNAT1 can directly repress the ET biosynthetic genes including *LcACS1/7* and *LcACO2/3*, to suppress ET production; (iii) LcKNAT1 can directly repress the transcription of *LcEIL2*/*3*, which are the central regulators of ET signaling; (iv) LcEIL2/3 can directly activate the transcription of *LcACS1*/*4*/*7* and *LcACO2*/*3* and cell wall degradation genes *LcCEL2*/*8* and *LcPG1*/*2*. Additionally, ET can repress *LcKNAT1* expression but promote *LcEIL2/3* transcription. Therefore, before abscission is triggered, *LcKNAT1* expression is maintained at a relatively high level, thereby resulting in a repressive state of both ET biosynthesis and signaling. In contrast, when *LcKNAT1* expression is inhibited by abscission-inducing signals, both the ET biosynthesis and signaling are derepressed, thereby activating the abscission process (Figure 7).

In the model plant *Arabidopsis*, genetic evidence has demonstrated that BP/KNAT1 functions in the control of the floral organ abscission by regulating the activity of two other KNOX-related genes *KNAT2* and *KNAT6* (Shi et al., 2011). In our previous study, a total of eight KNOX homologs were identified from the litchi genome. Interestingly, only LcKNAT1 displayed a regulatory function in the control of the floral organ abscission in *Arabidopsis* (Zhao et al., 2020). However, the overexpression of *KNAT2* or *KNAT6* only slightly promoted the floral organ abscission in *Arabidopsis* wild-type background (Shi et al., 2011), and it is possible that the overexpression of *LcKNAT3* or *LcKNAT6*, as LcKNAT3 and LcKNAT6 were the homologs to AtKNAT2 and AtKNAT6, displayed very weak phenotype. It will be interesting to further check whether the overexpression of *LcKNAT3* or *LcKNAT6* could also rescue the abscission phenotype in *ida* mutant. Thus, so far it could not be claimed that the mechanism underlying LcKNAT1 in control of the fruit abscission in litchi is different from that of BP/KNAT1 in *Arabidopsis*. Furthermore, we showed previously that KNAT1 binding motifs were also conserved in *Arabidopsis*; however, role of KNAT1 in regulating ET synthetic genes has never been investigated. It is likely that KNAT1 could negatively regulate both other KNOX members and the genes related to ET biosynthesis and signaling during the fruit abscission in litchi or floral organ abscission in *Arabidopsis*.

In *Arabidopsis*, BP/KNAT1 acts as the key downstream TF of IDA-HAE/HSL2 signaling to repress the floral organ abscission (Cho et al., 2008; Stenvik et al., 2008; Shi et al., 2011). Previously, we also found that the homologs of IDA and HAE/HSL2 are present in the litchi genome and demonstrated that LcIDL1 and LcHSL2 have similar functions in the control of the floral organ abscission in *Arabidopsis* (Ying et al., 2016; Wang et al., 2019). Additionally, since the ectopic expression of *LcKNAT1* decreases pedicel AZ activation in tomatoes (Zhao et al., 2020) and blocks floral organ abscission in *Arabidopsis* (Figure 5B), we can speculate that a conserved LcIDL1-LcHSL2 signaling module is probably sitting upstream and regulating the repressor activity of LcKNAT1. Interestingly, *ida* mutants could not be rescued by ET treatment, which led to the conclusion that the IDA-HAE/HSL2 pathway may control abscission in an ET-independent manner (Jinn et al., 2000; Butenko et al., 2003, 2006). However, this conclusion has been recently reconsidered with the findings that the IDA homologs from different species, including tomato, soybean, oil palm, citrus, and yellow lupine, are induced during the abscission process activated by ET (Tucker and Yang, 2012; Estornell et al., 2015; Stø et al., 2015; Wilmowicz et al., 2018). In litchi, *LcIDL1* and *LcHSL2* in the AZ are also ET-induced during the fruit abscission (Ying et al., 2016; Wang et al., 2019). Our results partially support the conclusion that the IDA-HAE/HSL2 abscission signaling module is ET-dependent (Botton and Ruperti, 2019; Meir et al., 2019) and propose that LcKNAT1 may act as the downstream of LcIDL1-LcHSL2 signaling subject to a no direct positive ET feedback mechanism and is required for the last steps of the execution of the abscission process. Thus, we hypothesized that ET treatment could have a dual effect by triggering the expression of genes encoding cell wall hydrolases and disabling the repressor function of LcKNAT1 through the indirect activation of the LcIDL1-LcHSL2 module *via* an unknown factor (Figure 7). In the future, efforts should be devoted to explore the role of ET in the AZ, in particular the cross talk between ET and IDA-HAE/HSL2 signaling pathways.

## Data Availability Statement

The authors acknowledge that the data presented in this study must be deposited and made publicly available in an acceptable repository, prior to publication. Frontiers cannot accept an article that does not adhere to our open data policies.

## Author Contributions

MZ and JL conceived and designed the experiments. XM performed most of the experiments. PY, ZH, and HW provided assistance. All authors contributed to the article and approved the submitted version.

## Conflict of Interest

The authors declare that the research was conducted in the absence of any commercial or financial relationships that could be construed as a potential conflict of interest.

## Publisher’s Note

All claims expressed in this article are solely those of the authors and do not necessarily represent those of their affiliated organizations, or those of the publisher, the editors and the reviewers. Any product that may be evaluated in this article, or claim that may be made by its manufacturer, is not guaranteed or endorsed by the publisher.
