# Corrigendum: The LcKNAT1-LcEIL2/3 regulatory module is involved in fruitlet abscission in litchi

**DOI:** 10.3389/fpls.2024.1413536

**Published:** 2024-04-30

**Authors:** Xingshuai Ma, Peiyuan Ying, Zidi He, Hong Wu, Jianguo Li, Minglei Zhao

**Affiliations:** ^1^ State Key Laboratory for Conservation and Utilization of Subtropical Agro-Bioresources, Guangdong Laboratory for Lingnan Modern Agriculture, Key Laboratory of Biology and Genetic Improvement of Horticultural Crops (South China), Ministry of Agriculture and Rural Affairs, Guangdong Litchi Engineering Research Center, College of Horticulture, South China Agricultural University, Guangzhou, China; ^2^ Guangdong Provincial Key Laboratory of Postharvest Science of Fruits and Vegetables, College of Horticulture, South China Agricultural University, Guangzhou, China

**Keywords:** litchi (*Litchi chinensis* Sonn.), fruitlet abscission, LcKNAT1, LcEIL2/3, ethylene

In the published article, there was an error in the affiliations. Instead of “1Guangdong Laboratory for Lingnan Modern Agriculture, Guangzhou, China

2State Key Laboratory for Conservation and Utilization of Subtropical Agro-Bioresources, Key Laboratory of Biology and Germplasm Enhancement of Horticultural Crops in South China at Ministry of Agriculture and Rural Affair, Guangdong Litchi Engineering Research Center, College of Horticulture, South China Agricultural University, Guangzhou, China

3Guangdong Provincial Key Laboratory of Postharvest Science of Fruits and Vegetables, College of Horticulture, South China Agricultural University, Guangzhou, China”, it should be

“^1^State Key Laboratory for Conservation and Utilization of Subtropical Agro-Bioresources, Guangdong Laboratory for Lingnan Modern Agriculture, Key Laboratory of Biology and Genetic Improvement of Horticultural Crops (South China), Ministry of Agriculture and Rural Affairs, Guangdong Litchi Engineering Research Center, College of Horticulture, South China Agricultural University, Guangzhou, China

^2^Guangdong Provincial Key Laboratory of Postharvest Science of Fruits and Vegetables, College of Horticulture, South China Agricultural University, Guangzhou, China”.

The authors apologize for this error and state that this does not change the scientific conclusions of the article in any way. The original article has been updated.

Published version:

**The LcKNAT1-LcEIL2/3 regulatory module is involved in fruitlet abscission in litchi**

Xingshuai Ma^1,2^, Peiyuan Ying^1^, Zidi He^1^, Hong Wu^1^, Jianguo Li^1,2,^* and Minglei Zhao^1,2,3,^*

^1^Guangdong Laboratory for Lingnan Modern Agriculture, Guangzhou, China

^2^State Key Laboratory for Conservation and Utilization of Subtropical Agro-Bioresources, Key Laboratory of Biology and Germplasm Enhancement of Horticultural Crops in South China at Ministry of Agriculture and Rural Affair, Guangdong Litchi Engineering Research Center, College of Horticulture, South China Agricultural University, Guangzhou, China

^3^Guangdong Provincial Key Laboratory of Postharvest Science of Fruits and Vegetables, College of Horticulture, South China Agricultural University, Guangzhou, China

^*^Corresponding author:

Jianguo Li, E-mail: jianli@scau.edu.cn

Minglei Zhao, E-mail: zml503@scau.edu.cn

Should be corrected to:

**The LcKNAT1-LcEIL2/3 regulatory module is involved in fruitlet abscission in litchi**

Xingshuai Ma^1^, Peiyuan Ying^1^, Zidi He^1^, Hong Wu^1^, Jianguo Li^1,2^* and Minglei Zhao^1,2^*

^1^State Key Laboratory for Conservation and Utilization of Subtropical Agro-Bioresources, Guangdong Laboratory for Lingnan Modern Agriculture, Key Laboratory of Biology and Genetic Improvement of Horticultural Crops (South China), Ministry of Agriculture and Rural Affairs, Guangdong Litchi Engineering Research Center, College of Horticulture, South China Agricultural University, Guangzhou, 510642, China

^2^Guangdong Provincial Key Laboratory of Postharvest Science of Fruits and Vegetables, College of Horticulture, South China Agricultural University, Guangzhou, China

