# Supplementary material for: The LcKNAT1-LcEIL2/3 Regulatory Module Is Involved in Fruitlet Abscission in Litchi
Source: Front Plant Sci. 2022 Jan 21;12:802016. doi: 10.3389/fpls.2021.802016 (PMC8813966; doi:10.3389/fpls.2021.802016)
Supplement: Supplementary file 1 [file Data_Sheet_1.doc]

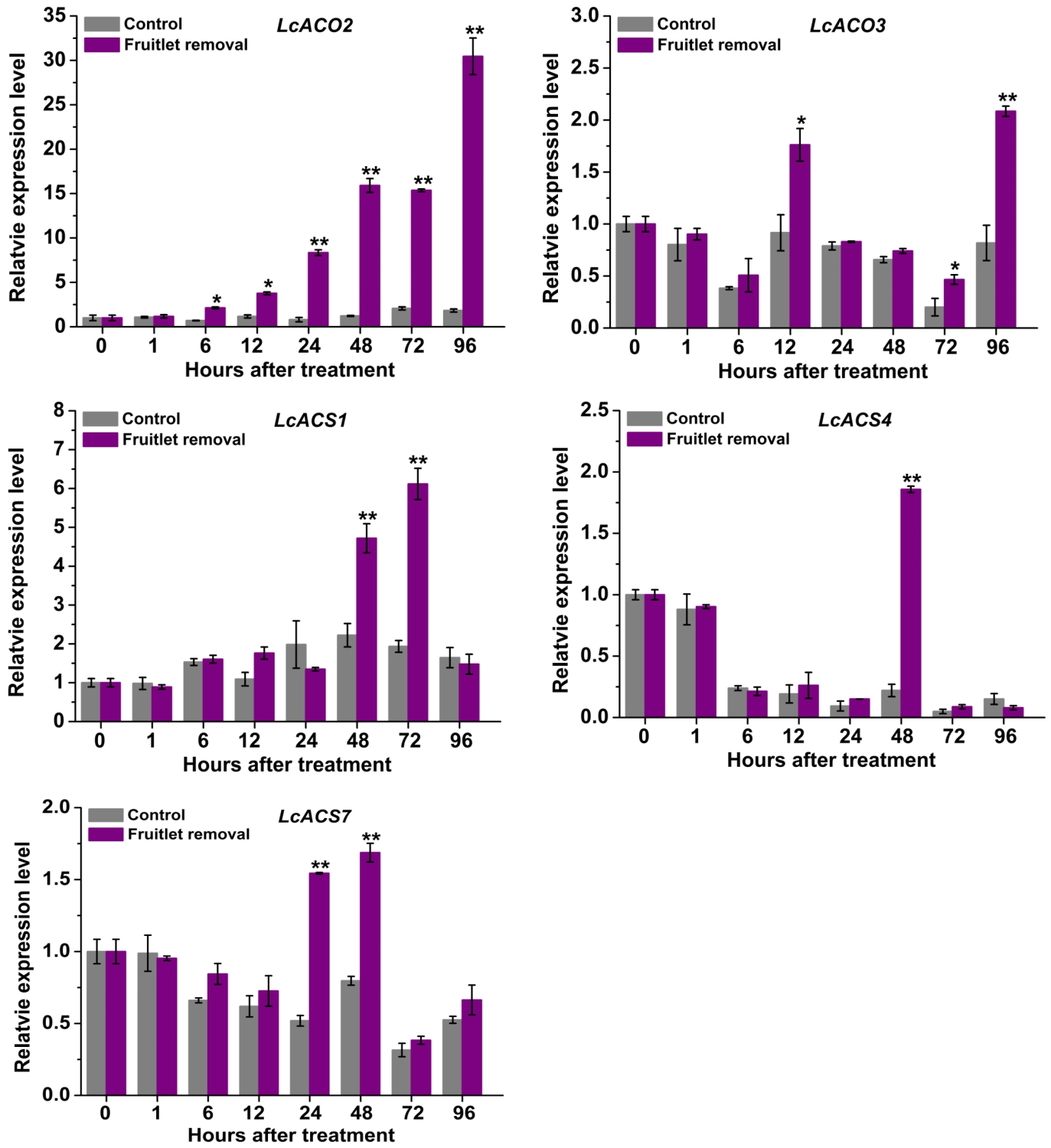


**Figure S1. The relative expression levels of *LcACO2/3*, and *LcACS1/4/7* in the FAZ during the fruitlet removal-induced fruitlet abscission in litchi.**

**Dataset S1 Nucleotide sequence of *LcEIL2* and *LcEIL3* promoters.**

**>*LcEIL2pro***

AGAACATTCACACTGACACATTCTATTTTAAAGAACAAAGTGAAACTTAAATTCTTAACTTCTTAAAGTCATATTTATTGGAGCTATTTAATCACTTTTATTTAGGGATAGTGTGCATTAATTAATGTATATAGTTTTATCGATTAAGATAGGCAAAAACAAGATATCGCACAAGAAAATGACAAAAATCCATCCCAGTTCCTGTCCATTAATAAATACGAAACATTATGTTGCATAAGAAAATGGCAAACATCCACCCTTATTCAAAATTTCACCCTAATTCTTGTCCATTGATAAATACAAAGCATTCTTAGGTAAGGCACATACAACCAAATTCCTATCCCTAGCAACAAATGAAGTTTTAAATAATGCAAAATTGAAAATACAGTTTAATAACTCCTAGTTGTTGTGCCAAGATGGCACAGCACACTTGCGCCTCATATGAGTACGCAAGTTCAAGTGTTGTCCATAGTAATACGGACAATTTTCGTTAGACTGCTCTTGTGTTAATGGACAAATTACGGAAAAGCACTTCAAGGTGGTAAGGATAATTTTCCTCTTAAATAGGGATTTGTGATCCAAGTCGCATCATAATGTCCATCATAGTGATTCTAGTCTTGGTAGATGTAATTAAGCTCAGAATAATAATAATAATAAGATTGTGTCCATTTTAATCATCAAATATAGTTACAATACTTTTGAAATAAGATTACCTAGCACATACATACTATTTGAATCTCACAATATTTTTAAAATTCAACTAATTACACCTCATTATTCTTTTAGTATAATATTTAGGATACATTTGTCAATAATACAAACGGATCCTAAATATTATAGGTATGAAGAGATCACTCTCCTATAGGTTTTAATGAAAGTTACAAATAACAAATATATATACATAAAAGTTAGCACTTATCTTGTTATGATAAATTGACGTGGATATCACCTCATGACAAATTATCTAATTCAAATTTCACACTGTAGTAGTTGTATTATATAACAAGGTCATATTACATGATATCAAGTTAGCACAAAATCACATAGCATGTTTTACTCATAAATGCTGCGGCGTGATGGCCACAGCACTTGCATCGTTTACAAACAGAAAATTATACATTTAAACTTCCAATAATTTAATTTAACTACAATATAACATCATGTATATATAACTATTAATATAAAATTCATGAAATACACAACGTTTTTAAATACAAATACAAAAGACTTGCTACATGCAAAATTTTCATTACAAATATCAAAAGACTTGTTGCATTTATAACAAAGTTTTATACATAATCCAAAATATTACTACTACATAGTCGTTCGAGATGGCAACGGATCGGATTGAATCTAGATCGATCCGTCCAAACCTGATTAGGAATTTTTGAAGGATGTCCTAAACCGACTCGTGCTTCAAACAGGTCTCTACTTTCTCCTCCAAACTTATGCCAAATAGATCGGGTTTCTCGGATTTAGATTTGATTGGGTATTTTTACCACCCCTAATGACAACCCACACTACATTGATACGGTAACTACAGTCCACATCTCTCGATATGGTAATTACAATATCTAAACATGCTTTTGGATACTTAATTTGGTCACAGCCGTTATGACTAGGTTCATGTCCAGCCATACATGCCCAAAATAAACAAGAGTCAAATAGACATGTATGGAAACTCCAAGGCAACAAAAGTCGAAGATGATATGCCCATAAAAAAAAAACAAAAAAGTTGAAGATGATATGATTTTAAAATAGACAAAACAGATCAAAACCCAGTAAGGCCACACAAATTATTTCATGTAGACGCAGCACAGGTGATCAAATCTCTCTAAAATAGCAGACTCCGATACGATTTGTATTTCTCCACCACTTGCAAAACTGCTTTTCGCCTCCTCCGAGCCTCTCTCTTTCTTCGAGATTTCTAGTTTCCAAATTATCGCATTTAATTGACCAAACTGTAATTATCTGTAAAAAATACAAAAAGGAAAAAAAATAACAAAACCCTTTTATGGGTGTGTGTTTCCTTTTCAGCGATTCATTTGTGTCTTATCTCTTGTAGCCAACTCTTTTCTCATTTGTATTTTTGGTCTGATAAGTCGATATAGAGATTGGAAAGTGAAAAGTGAGACGCCCCAGTTGGTGGGTTCGTGAAATTTGTCTCTGC**ATG**

**>*LcEIL3pro***

GTATCTATCAAGTGTCTTCTAAACACGTTAAATTATATTAAATAGGGAACAAAAATCTTTTATCAAGTTCAAGTTGATAGTTACGATTTTATACTTGCGTATGATACAACCAAAATACATACATACATATATATAGATATATATTAACAACATGTTATAATAGATCAAATGGTAAATAATACAAATGAACTTGAAAATGGTATTAACTTAAAAGAGGGTTAATATTGAAATGAATTTATTTTGTATATGATTCGTGTATGATAAGATGAGACGTTACAATAACGATATTAAATAAGACGATATGTCAAAATAGAAAGTGTTCTTTATGAAAAAAAGTTTAAAATGAATTTCTTCAAAGTATCTATAAATATTTTTTTTTAATATATTAAATAACAATGAAGTAAACGTTTCATTATGTTTGACCTCATAGTTTCATATCGATTATCGTATATAATTACAAGAAGAAACTTTGCATGTCGAACAATATGATCTTGTTAGAAGAAAAAAAATATATTCCTATGATCGATTGAATCCATGCACCCGAAACAAACTAAAACATTATCTTTCACAAATCCAAACTGAATCCATGTCAAACAATATGATCTTATTAGAATAAGTAACAATATTTCTACGATAGACCAAATCCATACACTTGAAACAAATTAAAACATTATCTTTTATAAACTCAAACCGAAACATTTTGTCACGTTCAAATGCAATAAAAAAAGGAAAAAAAGTAATATTACATTGTCAAAAACATTTTTTTTCCATATGATATTACGAACTATTAATTCATGGCAACGCTTCGATATTGTGAAATTGTGAAACGTAGCATTAATCAAAACAAAAAATAAAAAGAAAACAAAACCAATGCAGTGTAGTTGCCGGGTCACACGAGCAGTGAGTGATAGGTACAATAATTGGAGAATGAGAAAGAACACACAAGTGAGTCTCTCCTCTCTCTCTCTCTCTTCCAAAAAGTTACAAAAGGAAAGCAGCAAATCCATAAAACGCCAGAGAGAGACCCACGACTTAACCGCGGTGAAAAATAAACCTGAGACTAGGGTTATATACGACAACACTCTACAGTCTGACTCTCTTCATTCTTATCTCTCTCTCTCATTGCCATTTCTTCCTCAACTCTCCTACTACATAAACAAGTAACGCCAACGCAAACACAACAACAACATACTCTGTCTCTCTCTCTCTCTCTTCTTCTGTCTCTCTCTCGCGCGCGCGGGCGCGGTTTCAATGGATTATCCGATCAAGTGACTCTCTGGTATATCACTTCTCTCTCTCTCTATCTCTCTCTCTTCTCTTATATAATTGTTTTTCAGATTCTGTCTTATGTATCTCTTATCAGATGATCTTGATTATGTAGAATTCCATGCATGACACGCACAACACAGCCTTTCATGTCGTTTGTTGGGTGTCTCCGAATCTGATTTTGCTTTTTATTGGAACCATATGTATTCGGATTTGTAGTGATCGGATTGGTTTTTGTATATGTGGTCAAAAAGGTCACTTGTTTGCTAAATTAGAGAGGAATCTGGCTGTTTGATTGTTTGTTTGTGTTGAAAGTTGAATAAAAACAATTCCTTTGTGATTGTCTTTTTGCTAGTTTGTGTCAGGTCAGGCAGATCTGGGTTCTGGTGATTTTCTGCTCCTTTTCTGATTTATTTGATATTTATTTTGTGTTGTTTGTGATTTTGTTTTTGATCTTGAATTCTGAATATTAGATCAGTTTGTTGATTTGGACTTGCTTGAAACTCACTTTGGTGCTTTGGATAATCTATTGTTTTTTGTTTTTTTTCCTTTTATCATTTTGTGTGTTTGTTCTTGTTAATTATATCAATCTTTCATTTATGATGGTGCCATTACTTGCTCTATTAGTCTAATTTATGGCTTTTATGGTGATTACGTACTTGGTTTGTGTGTAATTGAATTGGAGTTATTATGGACTAGGGTTCGATGATTACGTATATGGTTTGAATGTTATTGAATTAGAGTTATGGACTAGGGTTCAATGTACCAGATATGGTATGTGGGAATTTTCTTATTTATTCATCAATTTTCAGGTGTAGAAAACTTGATCTGCGTTTCCGGGTTGATTTCCTGAGTTTTAAAAGTTATTGATATTTTGATCGCGAAGATCGGCAGGGAGTGAGAGAGGTTAGTCGTATTTGAAGTGAGTTTGATTTGGAATTGAATTGGGGTTTCGGTTTCTGTGACTGTGGGGAATGGGAATCTTTGAAGAA**ATG**

**Table S1 Summary of primers used in this study.**

| **Assay** | **Primer sequence (5’-3’)** | **Restriction Site** |
| --- | --- | --- |
| qRT-PCR | *LcKNAT1-qF*: TCAATCAAAGGAAACGCCACT  *LcKNAT1-qR*: TGCCCATTAACATCACCATCT  *GUS-qF*: GGTGGTGGCCAATGGTGATG  *GUS-qR*: GGCTTTTGGCTGTGACGCAC  *LcACO2-qF*: AAGTCAGTAACTACCCTCCATGCCCTAA  *LcACO2-qR*: GCCATCTGGTTGAGCAATCACACGG  *LcACO3-qF*: TGTCCTCATCCCGACCTTGTAAACG  *LcACO3-qR*: GGTAGCATTAACACGGTGCCAGAC  *LcACS1-qF*: CTGACCGCATTGTTATGAGTGGTGGAG  *LcACS1-qR*: GAGCCTTTTCATATGCAGCTTCCAA  *LcACS4-qF*: ACATATGGAGCCGCATTCACGTTG  *LcACS4-qR*: AGCACCTGATTCCTGCCGATTT  *LcACS7-qF*: CCAAGTAACAAGAGCAGCCGTAGAGGA  *LcACS7-qR*: TGGTGCCAAGTGGGTTTGAAGG  *AtUBQ-qF*: GATCTTTGCCGGAAAACAATTGGAGGATGGT  *AtUBQ-qR*: CGACTTGTCATTAGAAAGAAAGAGATAACAGG |  |
| Transgenic assay | *LcKNAT1-35SF:* ggactcttgaccatggta ATGGAAGGAGGATCTAGTGGTAGTACTAAT  *LcKNAT1-35SR:* gtcagatctaccatggt GAAGCGAGTTGGAGTGCAATCCATG | *Nco* Ⅰ |
| GUS assay | *Pro_LcEIL2_::GUS-F*: tcgacggatccccgggaattc CGGAAAAGCACTTCAAGGTGGTAAGG  *Pro_LcEIL2_::GUS-R*: gtggactcctcttagaattc GCAGAGACAAATTTCACGAACCCACC  *Pro_LcEIL3_::GUS-F*: tcgacggatccccgggaattc ACGATAGACCAAATCCATACACTTG  *Pro_LcEIL3_::GUS-R*: gtggactcctcttagaattc TTCTTCAAAGATTCCCATTCCCCAC | *Eco*R Ⅰ |
| Y1H assay | *pGADT7-LcKNAT1-F:* ggaggccagtgaattcATGGAAGGAGGATCTAGTGGTAGTACTAAT  *pGADT7-LcKNAT1-R:* cacccgggtggaattcGAAGCGAGTTGGAGTGCAATCCATG  *pAbAi-LcEIL2-F:* ttgaattcgagctcggtaccCTCGGATTTAGATTTGATTGGG  *pAbAi-LcEIL2-R:* atgcctcgaggtcgacGGGCATATCATCTTCGACTT  *pAbAi-LcEIL3-F:* ttgaattcgagctcggtaccGGAACCATATGTATTCGGAT  *pAbAi-LcEIL3-R:* atgcctcgaggtcgacGGAGCAGAAAATCACCAGAA | *Eco*R Ⅰ  *Kpn* Ⅰ  *Sal* Ⅰ |
| EMSA assay | *pGEX-4T-1-LcKNAT1-F*: gaattcccgggtcgac ATGGAAGGAGGATCTAGTGGTAGTACTAAT  *pGEX-4T-1-LKNAT1-R*: ggccgctcgagtcgac GAAGCGAGTTGGAGTGCAATCCATG  *LcEIL2-probe-F*: ACTTAATTTGGTCACAGCCGTTAtgactaggtTCATGTCCAGCCATACATGC  *LcEIL2-probe-R*: GCATGTATGGCTGGACATGAacctagtcaTAACGGCTGTGACCAAATTAAGT  *LcEIL2-mprobe-F*: ACTTAATTTGGTCACAGCCGTTAcccccccccTCATGTCCAGCCATACATGC  *LcEIL2-mprobe-R*: GCATGTATGGCTGGACATGAgggggggggTAACGGCTGTGACCAAATTAAGT  *LcEIL3-probe1-F*: GATTGTCTTTTTGCTAGTTTGTgtcaggtcaGGCAGATCTGGGTTCTGGTG  *LcEIL3-probe1-R*: CACCAGAACCCAGATCTGCCtgacctgacACAAACTAGCAAAAAGACAATC  *LcEIL3-mprobe1-F*: GATTGTCTTTTTGCTAGTTTGTcccccccccGGCAGATCTGGGTTCTGGTG  *LcEIL3-mprobe1-R*: CACCAGAACCCAGATCTGCCgggggggggACAAACTAGCAAAAAGACAATC  *LcEIL3-probe2-F*: GGATTGGTTTTTGTATATGTGgtcaaaaaggtcaCTTGTTTGCTAAATTAGAG  *LcEIL3-probe2-R*: CTCTAATTTAGCAAACAAGtgcctttttgacCACATATACAAAAACCAATCC  *LcEIL3-mprobe2-F*: GGATTGGTTTTTGTATATGTGtttttttttttttCTTGTTTGCTAAATTAGAG  *LcEIL3-mprobe2-R*: CTCTAATTTAGCAAACAAGaaaaaaaaaaaaaCACATATACAAAAACCAATCC | *Sal* Ⅰ |
| Dual-luciferase transient expression assay | *LcKNAT1-pGreenII 62-SK-F*: tagaactagtggatcc ATGGAAGGAGGATCTAGTGGTAGTACTAAT  *LcKNAT1-pGreenII 62-SK-R*: cggtatcgataagctt GAAGCGAGTTGGAGTGCAATCCATG  *Pro_LcEIL2_-LUC-F:* tatagggcgaattgg CGGAAAAGCACTTCAAGGTGGTAAGG  *Pro_LcEIL2_-LUC-R:* ttggcgtcttccatgg GCAGAGACAAATTTCACGAACCCACC  *Pro_LcEIL3_-LUC-F:* tatagggcgaattgg ACGATAGACCAAATCCATACACTTG  *Pro_LcEIL3_-LUC-R:* ttggcgtcttccatgg TTCTTCAAAGATTCCCATTCCCCAC | *Bam* HI  *Hind* III  *Kpn* Ⅰ  *Nco* Ⅰ |
